# Association of Depression With Precautionary Behavior Compliance, COVID-19 Fear, and Health Behaviors in South Korea: National Cross-sectional Study

**DOI:** 10.2196/42677

**Published:** 2023-02-22

**Authors:** Hyerine Shin, Ji-Su Kim, HyunHae Lee

**Affiliations:** 1 Department of Nursing Chung-ang University Seoul Republic of Korea

**Keywords:** COVID-19, precautionary behaviors, COVID-19 fear, health behavior deterioration, gender differences

## Abstract

**Background:**

As of January 2022, the number of people infected with COVID-19 worldwide has exceeded 350 million. As the COVID-19 pandemic continues, people are affected in a wide range of areas of life, which in turn causes numerous psychological problems. Depression is a serious problem for people who have suffered from COVID-19. Depression can worsen COVID-19 precautionary behavior compliance or the health behavior itself. In addition, these depressive symptoms may have different characteristics depending on the individual’s gender.

**Objective:**

The aim of this study was to determine whether depression is a factor that may affect COVID-19 fear, precautionary behavior compliance, and health behavior, and how these characteristic trends differ by gender.

**Methods:**

This was a secondary analysis of data from the 2020 Korea Community Health Survey (KCHS), a national cross-sectional survey conducted with complex sampling analysis. In 2020, the KCHS included COVID-19–related questions. For this study, we used the KCHS data from both the COVID-19–related questions and the Patient Health Questionnaire-9 scale. After weighting the data according to the KCHS guidelines, we calculated the distribution of men and women according to depression level. The data were collected using multiple-choice questions related to precautionary behavior compliance, COVID-19–related fears, and health behavior changes.

**Results:**

Of the 204,787 participants, those who were clinically depressed had a greater tendency to not comply with precautionary behaviors. Regarding COVID-19, “fear” showed a decreasing trend in both men (adjusted odds ratio [AOR] 0.72, 95% CI 0.61-0.83) and women (AOR 0.74, 95% CI 0.63-0.86) with clinically relevant depression. Moreover, for both men and women, health behaviors deteriorated as depression intensified; the AOR for sleep duration changes was 2.28 (95% CI 2.00-2.59) in men and was 2.15 (95% CI 1.96-2.36) in women. Notably, the responses of clinically depressed women revealed a doubled increase in both their drinking (AOR 2.25, 95% CI 1.88-2.70) and smoking (AOR 2.71, 95% CI 1.95-3.77) habits compared with those of nondepressed women.

**Conclusions:**

Both men and women with more severe depression were more likely to violate precautionary health behaviors as their depression worsened. Health behaviors also deteriorated for both genders, but women tended to show a greater change. Therefore, additional studies and interventions for vulnerable groups such as severely depressed people are needed. More research is also necessary to develop interventions based on statistical comparisons of men and women.

## Introduction

As of January 25, 2022, 349,641,119 people worldwide had been infected with COVID-19 and the death toll had reached 5,592,266 [1]. Since the World Health Organization’s March 11, 2020, declaration that the world was facing a pandemic, various restrictions have been implemented to prevent the global spread of the virus. These restrictions have included lockdowns for all or some educational facilities and nonessential businesses, travel limitations, quarantine measures, social isolation, and mandatory mask-wearing [2-4]. However, COVID-19 has had a profound impact on all countries, racial groups, and socioeconomic classes [5]. Secondary problems arise from these restrictions.

Among all the problems that have occurred, mental health issues related to COVID-19 are among the most important health concerns and have received considerable attention. Direct stress caused by illness and concerns about medical service interruption, employment, financial stability, and social contact restrictions may cause psychological problems [6-9]. Additionally, COVID-19 fear is associated with several mental health problems such as anxiety, depression, psychological distress, and sleep issues [10-12]. In particular, depression is one of the most representative mental health problems during COVID-19, which is associated with deterioration in health behaviors, including decreased physical activity and sleep and increased smoking [13-15].

Moreover, various empirical studies have reported gender differences in depression associated with the COVID-19 pandemic [16-18]. Depression is known to have some gender-specific characteristics, and depression rates are generally higher in women regardless of COVID-19 [19,20]. For example, among Australian adults, women were found to be more likely to have clinically significant depression and anxiety [17]. In the case of COVID-19, these differences still apply, as indicated by a study of adults aged 25-69 years showing that women reported more depression, anxiety, stress, loneliness, and a higher rate of increased cannabis use compared to pre-COVID-19 levels [16].

Mental health is not only a concern in and of itself, but may also impede health behaviors or COVID-19 precautionary actions [15,21,22]. Olapegba et al [23] reported that psychological distress caused by COVID-19 fear hinders precautionary behavior compliance. Moreover, depression can lead to even more significant health problems by reducing COVID-19 precautionary behavior compliance and general healthy behavior. In addition, given that severe degrees of fear can negatively impact an individual’s ability to respond appropriately during the COVID-19 pandemic [24], the impact of depression may vary in severity, and, at the same time, gender differences may also exist. To date, studies have been conducted on the gender gap in depression related to COVID-19 or the following problems: medical service interruption, employment, financial stability, social contact restrictions [25-27], and the impact of depression on COVID-19 precautionary behavior compliance [22,28]. However, studies that have analyzed the severity of depression according to gender are scarce. Therefore, the purpose of this study was to determine whether depression is a factor that worsens COVID-19 fears, precautionary behavior compliance, and health behavior, and whether these characteristic trends differ by gender.

## Methods

### Study Design

This was a secondary analysis of data from a national cross-sectional survey: the 2020 Korea Community Health Survey (KCHS) conducted by the Korea Centers for Disease Control and Prevention [29]. The Community Health Survey, initiated in 2008, is an annual, anonymous, computer-assisted personal interview survey that evaluates the health status and behaviors of South Korean adults. A trained surveyor visits the households selected as samples and conducts a one-on-one interview using a laptop computer equipped with a survey program. The 2020 Community Health Survey covered 18 geographical areas and comprised 142 survey questions, including those pertaining to health behaviors such as exercise, smoking, and drinking. Given the global health context in 2020, the survey for that year was revised to include questions related to the COVID-19 pandemic.

The data were collected from August 16, 2020, to October 31, 2020 [29]. The target population of 2020 KCHS was adults aged 19 years or older, and a sample group was extracted through a composite sample design to minimize sample bias [30]. Next, in the first official extraction, probability proportional to size sampling was performed to ensure that the extraction probability was proportional to the number and size of households by housing type in Tong, Ban, and Ri (the smallest administrative district units). The second extraction was a systematic extraction based on the number of households in Tong, Ban, and Ri. In total, 229,269 people were surveyed; after excluding incomplete responses, the data from 204,787 respondents were available for analysis. To request the KCHS data for use in the present study, we signed a written pledge to confirm our intentions and submitted a usage plan describing the purpose for which we intended to use the data if, after the Korea Centers for Disease Control and Prevention reviewed the plan, the data request was approved. Only those who have received approval can download and use the raw data of KCHS.

### Ethical Approval

This study was conducted after review from the Institutional Review Board (IRB) of Chung-ang University. Because this was a secondary analysis of data from a national cross-sectional survey, the study received an exemption by the IRB (approval number 1041078-202201-HR-002).

### Measures

#### General Participant Characteristics

To determine the participants’ general characteristics, we included data about their sociodemographic characteristics and individual health. Sociodemographic characteristics were assessed according to age, gender, educational level, monthly household income, profession, and region. Age was divided into four generational groups based on the criteria used in the Korean Longitudinal Study of Aging and previous studies [31-33]: 20 to 44 years (youth), 45 to 64 years (middle age), and 65 years or older (older adults). According to a previous study, education levels were categorized as follows: (1) elementary or less, (2) middle school, (3) high school, and (4) college or higher. Monthly household income was classified into quartiles as Q1 (<25%), Q2 (25%-49%), Q3 (50%-74%), and Q4 (≥75%), and professions were organized according to the Korean version of the Standard Classification of Occupations and previous studies [34,35]. After referring to previous research, we reclassified occupations into four categories: white (white collar), pink (sales and service), blue (agricultural, forestry, fishing, and military), and unemployed. Region was divided into urban and rural areas.

Questions related to individual health conditions included drinking at least once a month, current smoking status, stress awareness, and subjective health status. Possible answers for drinking at least once per month, current smoking status, and stress awareness were either “yes” or “no.” Subjective health status was defined by the self-evaluation of one’s health as “very good” or “good,” and possible answers were “poor” and “fair.”

#### Depression Assessment

The Patient Health Questionnaire-9 (PHQ-9) was used to measure self-reported depression, with possible scores ranging from 0 to 27 [35]. PHQ-9 consists of nine items used to evaluate the diagnostic criteria for major depressive disorder in accordance with the Diagnostic and Statistical Manual of Mental Disorders, fifth edition. A high PHQ-9 score indicates a higher level of depressive symptoms, with 0-4 representing no depression, 5-9 indicating mild depression, 10-14 representing moderate depression, 15-19 indicating moderately severe depression, and 20-27 representing severe depression. In general, a PHQ-9 score ≥10 indicates major or clinically relevant depression [36]. To ensure greater clarity in the explanation of the results, we divided the PHQ-9 scores into three categories: 0-4 (none), 5-9 (mild), and ≥10 (clinically relevant).

#### Precautionary Behavior Compliance

Precautionary behavior compliance was defined as whether personal quarantine rules (eg, wearing a mask indoors, wearing a mask outdoors) and social distancing rules (eg, maintaining a healthy distance) were observed for the past week with the intention of helping to prevent the spread of COVID-19. According to a previous study [37], eight factors were used to evaluate precautionary behavior compliance as follows: in the last 7 days, had the individual (1) covered his or her mouth and nose while sneezing or coughing, (2) experienced daily ventilation at least twice a day, (3) had everyday spaces disinfected once per day, (4) wore a mask in facilities used by unspecified people, (5) worn a mask when it was impossible to keep an adequate distance, (6) maintained a 2-meter distance between themselves and others, (7) limited visiting sick people, and (8) limited going out or attending gatherings and events? Based on the KCHS’s indicator definition, for each of the eight items, only those who answered “very much” or “yes” were considered to have complied with each precautionary behavior. For example, only the respondents who answered “very much” or “yes” to the question “covered his or her mouth and nose while sneezing or coughing” were considered to have performed precautionary behavior.

#### COVID-19 Fear

COVID-19 fear is defined as a psychological concern due to the spread of COVID-19 and is assessed by five factors: fear of infection, dying from infection, public criticism, a family member getting infected, and economic loss due to infection. Based on the KCHS’s indicator definition, only those who answered “strongly agree” or “agree” for any of the above five factors were evaluated as having COVID-19 fear of each factor.

#### Health Behavior Deterioration

Criteria pertaining to physical activity, sleep duration, fast food or energy drink consumption, food consumption, alcohol consumption, and smoking were included to investigate health behavior deterioration during the COVID-19 pandemic. Possible responses were “increased,” “same,” “decreased,” or “not applicable.” With reference to a previous study [37], health behavior deterioration was measured as follows: decreased physical activity, changes in sleep duration (either increased or decreased), increased consumption of instant meals/soda, increased consumption of delivery food, increased alcohol consumption, and an increase in the number of cigarettes smoked per day.

### Data Analysis

The distribution of men and women according to depression level was calculated after weighting based on the KCHS guidelines. The weighted values of the distribution of men and women according to depression level are shown as descriptive statistics such as frequency, percentage, and standard error. The participants’ general characteristics according to depression level and gender were also analyzed using descriptive statistics such as frequency, percentage, and standard error. We performed a *χ*^2^ test to confirm the general distribution of the characteristics according to the depression level of the participants.

Depression levels, gender-specific precautionary behavior noncompliance, COVID-19 fear, and health behavior deterioration were analyzed using logistic regression. After controlling for general characteristics (age, educational level, monthly household income, profession, region, heavy drinking, current smoking status, stress awareness, and subjective health status), logistic regression analysis was used to identify differences in depression level and gender-specific precautionary behavior noncompliance, COVID-19 fear, and health behavior deterioration. The adjusted odds ratios (AORs) and 95% CIs for failure to comply with precautionary behaviors, COVID-19–related fear, and health behavior deterioration were calculated. All results are presented as weighted values. The results were statistically analyzed using SPSS version 26.0 (IBM Corp, Armonk, NY, USA).

## Results

Table 1 shows the weighted and unweighted distributions of depression in the population according to gender. This study included 204,787 participants (92,739 men and 112,048 women). Approximately 2% and 3.5% of men and women were classified as having clinically relevant depression, respectively.

Table 2 shows the participants’ general characteristics, and Table 3 and Table 4 further summarize these data by depression levels for men and women, respectively. To better represent the general population of South Korea, the percentages and standard errors are presented as weighted values. The largest group of men with clinically relevant depression (48.8%) were aged 20-44 years, and 44.6% of men with clinically relevant depression had a college degree or higher. Approximately 41.6% belonged to the income level Q1, and the rate of unemployment reached 45.5%. Although there was no significant difference in the level of mental health by region, 45.6% of the men with clinically relevant depression reported heavy drinking and 44.5% were current smokers. Among the men with clinically relevant depression, only 21.3% answered that they had stress, but 79.3% evaluated their subjective health condition as poor. Among the women with clinically relevant depression, 60.3% were unemployed. Notably, self-reported heavy drinking increased to 19.4% and current smoking increased to 11.7%, which was five times higher than that of women without depression symptoms

**Table 1:** Overall and weighted distribution of depression in the sample population (N=204,787).

Depression level^a^			Unweighted, n (%)		Weighted^b^		
					Participants, n (%)		SE
**Men (n=92,739)**							
	None (0-4)	83,026 (89.5)		16,832,027 (88.4)		0.1	
	Mild (5-9)	7889 (8.5)		1,799,468 (9.5)		0.1	
	Clinically relevant (≥10)	1824 (2.0)		399,490 (2.1)		0.1	
**Women (n=112,048)**							
	None (0-4)	93,305 (83.3)		15,943,821 (82.3)		0.2	
	Mild (5-9)	14,771 (13.2)		2,683,383 (13.9)		0.1	
	Clinically relevant (≥10)	3972 (3.5)		736,847 (3.8)		0.1	

^a^Classified according to Patient Health Questionnaire-9 scores.

^b^Data were weighted to yield nationally representative estimates (total N=38,395,036).

**Table 2:** Participants’ general characteristics (N=204,787).

Characteristics		Unweighted, n	Weighted^a^	
			Participants, n	% (SE)
**Age (years)**				
	20-44	60,924	16,207,118	42.2 (0.1)
	45-64	77,873	14,448,396	37.6 (0.1)
	65 and over	65,990	7,739,521	20.2 (0.1)
**Educational level**				
	Elementary or less	45,804	4,453,550	11.6 (0.1)
	Middle school	23,051	3,089,900	8.1 (0.1)
	High school	58,976	11,267,036	29.3 (0.1)
	College or higher	76,956	19,584,549	51.0 (0.2)
**Monthly household income quartile (Q)**				
	Q1 (lowest)	72,643	9,499,542	24.7 (0.1)
	Q2	51,997	9,871,080	25.7 (0.1)
	Q3	41,606	9,416,797	24.5 (0.1)
	Q4 (highest)	38,541	9,607,616	25.0 (0.2)
**Profession^b^**				
	White	38,617	9,999,414	26.0 (0.1)
	Pink	25,798	5,195,447	13.5 (0.1)
	Blue	59,749	8,538,958	22.2 (0.1)
	None	80,623	14,661,216	38.2 (0.1)
**Region**				
	Urban	58,299	16,465,931	42.9 (0.1)
	Rural	146,488	21,929,104	57.1 (0.1)
**Heavy drinking**				
	Never	86,787	13,072,593	34.0 (0.1)
	No	67,789	13,778,747	35.9 (0.1)
	Yes	50,211	11,543,695	30.1 (0.1)
**Current smoking status**				
	Never	134,123	24,290,719	63.3 (0.1)
	Former	37,613	7,183,170	18.7 (0.1)
	Current	33,051	6,921,146	18.0 (0.1)
**Stress awareness**				
	No	45,478	9,869,519	25.7 (0.1)
	Yes	159,309	28,525,516	74.3 (0.1)
**Subjective health status**				
	Poor	107,046	18,202,506	47.4 (0.1)
	Fair	97,741	20,192,529	52.6 (0.1)

^a^Data were weighted to yield nationally representative estimates (total N=38,395,036).

^b^Classified into white (white collar), pink (sales and service), blue (agricultural, forestry, fishing, and military), and none (unemployed).

**Table 3:** Comparison of men’s general characteristics by depression level (N=92,739).

Characteristics			No depression (PHQ-9^a^ 0-4)					Mild depression (PHQ-9 5-9)					Clinically relevant depression (PHQ-9≥10)					*F* ^b^				*df*				*P* value	
			Unweighted^c^, n		Weighted^d^, n (%, SE)		Unweighted, n			Weighted, n (%, SE)		Unweighted, n			Weighted, n (%, SE)												
**Age (years)**																			26.63				3.88, 61,102.47				<.001
	20-44	25,820		7,320,210 (43.5, 0.2)		2762			875,763 (48.7, 0.7)		614			194,869 (48.8, 1.5)													
	45-64	32,700		6,460,358 (38.4, 0.2)		2639			595,349 (33.1, 0.7)		561			116,955 (29.3, 1.3)													
	65 and over	24,506		3,051,459 (18.1, 0.2)		2488			328,356 (18.2, 0.5)		649			87,666 (21.9, 1.1)													
**Educational level**																			27.64				5.73, 90,089.21				<.001
	Elementary or less	10,998		1,075,442 (6.4, 0.1)		1340			144,948 (8.1, 0.3)		418			51,493 (12.9, 0.8)													
	Middle school	9269		1,194,121 (7.1, 0.1)		930			138,617 (7.7, 0.3)		250			40,617 (10.2, 0.8)													
	High school	26,371		5,020,817 (29.8, 0.2)		2347			549,575 (30.5, 0.6)		555			129,132 (32.3, 1.4)													
	College or higher	36,388		9,541,647 (56.7, 0.2)		3272			966,328 (53.7, 0.7)		601			178,248 (44.6, 1.5)													
**Monthly household income quartile (Q)**																			62.22				5.93, 93,308.82				<.001
	Q1 (lowest)	25,438		3,589,381 (21.3, 0.2)		3115			505,037 (28.1, 0.6)		949			166,331 (41.6, 1.4)													
	Q2	21,809		4,321,547 (25.7, 0.2)		1864			459,319 (25.5, 0.6)		391			94,942 (23.8, 1.4)													
	Q3	18,316		4,347,118 (25.8, 0.2)		1534			422,973 (23.5, 0.6)		257			72,562 (18.2, 1.2)													
	Q4 (highest)	17,463		4,573,982 (27.2, 0.2)		1376			412,139 (22.9, 0.6)		227			65,655 (16.4, 1.1)													
**Profession^e^**																			42.96				5.94, 93,506.52				<.001
	White	17,865		4,938,141 (29.3, 0.2)		1533			470,702 (26.2, 0.7)		251			84,165 (21.1, 1.3)													
	Pink	8371		2,013,564 (12.0, 0.2)		819			229,551 (12.8, 0.5)		165			45,913 (11.5, 1.0)													
	Blue	34,082		5,513,592 (32.8, 0.2)		2737			558,664 (31.0, 0.7)		464			87,410 (21.9, 1.2)													
	None	22,708		4,366,730 (25.9, 0.2)		2800			540,551 (30.0, 0.6)		944			182,002 (45.5, 1.5)													
**Region**																			1.05				1.99, 31,436.14				.35
	Urban	23,214		7,100,130 (42.2, 0.2)		2438			778,683 (43.3, 0.7)		559			169,067 (42.3, 1.5)													
	Rural	59,812		9,731,897 (57.8, 0.2)		5451			1,020,785 (56.7, 0.7)		1265			230,423 (57.7, 1.5)													
**Heavy drinking**																			16.41				3.98, 62,618.59				<.001
	Never	24,042		3,914,812 (23.3, 0.2)		2416			418,786 (23.3, 0.6)		725			127,952 (32.0, 1.3)													
	No	24,751		5,072,726 (30.1, 0.2)		2202			514,892 (28.6, 0.7)		405			89,483 (22.4, 1.2)													
	Yes	34,233		7,844,489 (46.6, 0.2)		3271			865,790 (48.1, 0.7)		694			182,055 (45.6, 1.5)													
**Smoking status**																			53.93				3.98, 62,603.76				<.001
	Never	25,752		5,621,708 (33.4, 0.2)		1914			474,901 (26.4, 0.6)		423			93,702 (23.4, 1.5)													
	Former	30,840		5,750,630 (34.2, 0.2)		3004			613,040 (34.1, 0.7)		654			128,119 (32.1, 1.3)													
	Current	26,434		5,459,689 (32.4, 0.2)		2971			711,527 (39.5, 0.7)		747			177,669 (44.5, 1.4)													
**Stress awareness**																			2598.88				1.99, 31,378.61				<.001
	No	14,374		3,431,190 (20.4, 0.2)		3997			1,021,365 (56.8, 0.7)		1342			314,591 (78.7, 1.2)													
	Yes	68,652		13,400,837 (79.6, 0.2)		3892			778,103 (43.2, 0.7)		482			84,899 (21.3, 1.2)													
**Subjective health status**																			867.4				2.00, 31,446.08				<.001
	Poor	36,364		6,707,215 (39.8, 0.2)		5450			1,150,997 (64.0, 0.7)		1510			316,919 (79.3, 1.2)													
	Fair	46,662		10,124,812 (60.2, 0.2)		2439			648,471 (36.0, 0.7)		314			82,571 (20.7, 1.2)													

^a^PHQ-9: Patient Health Questionnaire-9.

^b^Rao-Scott composite sample *χ*^2^ test.

^c^Data were not weighted (total N= 204,787).

^d^Data were weighted to yield nationally representative estimates (total N=38,395,036).

^e^Classified into white (white collar), pink (sales and service), blue (agricultural, forestry, fishing, and military), and none (unemployed).

**Table 4:** Comparison of women’s general characteristics by depression level (N=112,048).

Characteristics			No depression (PHQ-9^a^ 0-4)					Mild depression (PHQ-9 5-9)					Clinically relevant depression (PHQ-9≥10)					*F* ^b^				*df*				*P* value	
			Unweighted^c^, n		Weighted^d^, n (%, SE)		Unweighted, n			Weighted, n (%, SE)		Unweighted, n			Weighted, n (%, SE)												
**Age (years)**																			64.38				3.91, 62,134.61				<.001
	20-44	26,185		6,354,538 (39.8, 0.2)		4323			1,137,258 (42.4, 0.5)		1220			324,480 (44.0, 1.0)													
	45-64	36,214		6,187,323 (38.8, 0.2)		4674			880,246 (32.8, 0.5)		1085			208,165 (28.3, 0.9)													
	65 and over	30,906		3,401,959 (21.3, 0.2)		5774			665,878 (24.8, 0.4)		1667			204,202 (27.7, 0.8)													
**Educational level**																			41.03				5.81, 92,331.79				<.001
	Elementary or less	26,408		2,480,195 (15.6, 0.1)		5111			528,808 (19.7, 0.4)		1529			172,665 (23.4, 0.7)													
	Middle school	10,646		1,411,759 (8.9, 0.1)		1563			234,528 (8.7, 0.3)		393			70,257 (9.6, 0.6)													
	High school	25,060		4,590,105 (28.8, 0.2)		3635			759,285 (28.3, 0.5)		1008			218,122 (29.6, 0.9)													
	College or higher	31,191		7,461,761 (46.8, 0.2)		4462			1,160,761 (43.3, 0.5)		1042			275,803 (37.4, 1.0)													
**Monthly household income quartile (Q)**																			95.29				5.94, 94,443.28				<.001
	Q1 (lowest)	34,270		4,045,405 (25.4, 0.2)		6709			876,383 (32.7, 0.5)		2162			317,006 (43.0, 1.0)													
	Q2	23,614		4,117,698 (25.8, 0.2)		3483			697,517 (26.0, 0.5)		836			180,058 (24.4, 0.9)													
	Q3	18,471		3,866,590 (24.3, 0.2)		2481			579,397 (21.6, 0.5)		547			128,155 (17.4, 0.8)													
	Q4 (highest)	16,950		3,914,127 (24.5, 0.2)		2098			530,085 (19.8, 0.5)		427			111,628 (15.1, 0.8)													
**Profession^e^**																			36.44				5.90, 93,721.40				<.001
	White	16,222		3,802,298 (23.8, 0.2)		2277			577,758 (21.5, 0.5)		469			126,349 (17.1, 0.8)													
	Pink	14,037		2,420,483 (15.2, 0.2)		1926			380,193 (14.2, 0.4)		480			105,743 (14.3, 0.7)													
	Blue	19,458		2,021,899 (12.7, 0.1)		2520			296,602 (11.1, 0.3)		488			60,792 (8.3, 0.5)													
	None	43,588		7,699,140 (48.3, 0.2)		8048			1,428,829 (53.2, 0.5)		2535			443,963 (60.3, 1.0)													
**Region**																			0.049				1.99, 31,747.28				.95
	Urban	26,358		6,928,218 (43.5, 0.2)		4522			1,167,254 (43.5, 0.5)		1208			322,577 (43.8, 1.0)													
	Rural	66,947		9,015,602 (56.5, 0.2)		10,249			1,516,128 (56.5, 0.5)		2764			414,270 (56.2, 1.0)													
**Heavy drinking**																			56.69				3.98, 63,305.92				<.001
	Never	49,462		7,109,633 (44.6, 0.2)		7909			117,411 (43.8, 0.5)		2233			326,999 (44.4, 1.0)													
	No	34,385		6,794.290 (42.6, 0.2)		4900			1,040,293 (38.8, 0.5)		1146			267,063 (36.2, 1.0)													
	Yes	9458		2,039,897 (12.8, 0.2)		1962			468,678 (17.4, 0.4)		593			142,785 (19.4, 0.8)													
**Smoking status**																			350.97				3.97, 63,128.55				<.001
	Never	89,302		1,513,9982 (95.0, 0.1)		13,416			2,371,959 (88.4, 0.3)		3316			588,466 (79.9, 0.8)													
	Former	2120		455,587 (2.8, 0.1)		714			173,855 (6.5, 0.3)		281			61,940 (8.4, 0.6)													
	Current	1883		348,251 (2.2, 0.1)		641			137,568 (5.1, 0.2)		375			86,441 (11.7, 0.6)													
**Stress awareness**																			3865.37				1.99, 31,722.31				<.001
	No	15,760		3,131,642 (19.6, 0.2)		7157			1,413,307 (52.7, 0.5)		2848			557,423 (75.6, 0.8)													
	Yes	77,545		1,281,2178 (80.4, 0.2)		7614			1,270,075 (47.3, 0.5)		1124			179,424 (24.4, 0.8)													
**Subjective health status**																			1144.07				1.99, 31,746.02				<.001
	Poor	49,326		7,556,843 (47.4, 0.2)		11,041			1,874,240 (69.8, 0.5)		3355			596,292 (80.9, 0.8)													
	Fair	43,979		8,386,977 (52.6, 0.2)		3730			809,142 (30.2, 0.5)		617			140,555 (19.1, 0.8)													

^a^PHQ-9: Patient Health Questionnaire-9.

^b^Rao-Scott composite sample *χ*^2^ test.

^c^Data were not weighted (total N= 204,787).

^d^Data were weighted to yield nationally representative estimates (total N=38,395,036).

^e^Classified into white (white collar), pink (sales and service), blue (agricultural, forestry, fishing, and military), and none (unemployed).

Table 5 illustrates how compliance with precautionary behaviors, COVID-19 fear, and health behavior deterioration differed according to gender and mental health determined using logistic regression analysis after adjusting for general characteristics. The crude odds ratios from the unadjusted model are shown in Multimedia Appendix 1. The precautionary behavior noncompliance rate was found to increase as the depression worsened; this was the case for most items compared to the group without depressive symptoms. The AOR of the noncompliance rate regarding wearing a mask indoors was lower in participants with mild depressive symptoms compared to that for participants with clinically relevant depression, which was the case for both men and women (Table 5). However, in the case of noncompliance with precautionary behaviors related to social distancing, the trend was different between men and women. Among men who had mild depressive symptoms, nonobservance of maintaining physical distance increased by more than that among men with clinically relevant depression. By contrast, the AOR of not maintaining physical distance in women was higher for those with clinically relevant depression than for those with mild depression, showing a tendency to worsen as mental health deteriorated.

The data also showed different characteristics of COVID-19 fear between men and women. The AOR of the fear of infection was similar for women and men with clinically relevant depression. However, the fear of death due to infection was significant only in women for those with mild depressive symptoms. In the case of public criticism, a significant association appeared only among women with clinically relevant depression.

As depression increased in severity in both men and women, health behaviors likewise deteriorated. Changes in sleep duration, an increase in drinking amount, and smoking frequency more than doubled for both men and women with clinically relevant depression. Regarding delivery food consumption, the AOR was the highest for men with mild depressive symptoms, which was similar to that for women with clinically relevant depression.

**Table 5:** Adjusted odds ratios (95% CIs) for failure to comply with precautionary behaviors, health behavior deterioration during the COVID-19 outbreak, and COVID-19–related fear according to depression levels.^a,b,c^

COVID-19–related questions		Men (n=92,739)				Women (n=112,048)		
		No depression^d^ (0-4)	Mild depression (5-9)	Clinically relevant depression (≥10)	No depression^d^ (0–4)		Mild depression (5–9)	Clinically relevant depression (≥10)
**Failure to comply with precautionary behaviors**								
	Not covering mouth while coughing	1	*1.29 (1.13-1.46)* ^e^	*1.56 (1.26-1.93)*	1		*1.38 (1.24-1.55)*	*1.65 (1.40-1.95)*
	No proper ventilation	1	*1.54 (1.29-1.83)*	*1.73 (1.28-2.34)*	1		*1.59 (1.34-1.90)*	*2.22 (1.75-2.81)*
	Not performing regular disinfection	1	*1.23 (1.16-1.31)*	*1.32 (1.16-1.50)*	1		*1.19 (1.13-1.25)*	*1.24 (1.13-1.36)*
	Not wearing a mask indoors	1	*1.54 (1.04-2.27)*	*1.81 (1.07-3.06)*	1		*1.49 (1.02-2.18)*	*2.04 (1.21-3.44)*
	Not wearing a mask when it was hard to maintain distance	1	*1.44 (1.12-1.85)*	1.42 (0.90-2.23)	1		*1.57 (1.21-2.02)*	*1.93 (1.35-2.77)*
	Not keeping the minimum recommended physical distance	1	*1.41 (1.23-1.61)*	*1.34 (1.03-1.74)*	1		*1.49 (1.33-1.67)*	*1.57 (1.29-1.90)*
	Not refraining from visiting hospitalized patients	1	*1.82 (1.41-2.35)*	*1.89 (1.14-3.13)*	1		*1.29 (1.02-1.62)*	1.35 (0.93-1.94)
	Not refraining from going out	1	*1.55 (1.31-1.85)*	*1.51 (1.13-2.00)*	1		*1.56 (1.35-1.81)*	*1.36 (1.06-1.75)*
**COVID-19–related fears**								
	Fear of infection	1	0.96 (0.87-1.05)	*0.72 (0.61-0.86)*	1		1.05 (0.95-1.15)	*0.74 (0.63-0.86)*
	Fear of dying from infection	1	1.05 (0.98-1.12)	1.07 (0.94-1.22)	1		*1.09 (1.03-1.15)*	1.04 (0.94-1.14)
	Fear of public criticism	1	0.95 (0.88-1.04)	0.85 (0.72-1.00)	1		1.07 (0.98-1.16)	*0.86 (0.75-0.99)*
	Fear of a family member getting infected	1	1.06 (0.92-1.22)	0.83 (0.66-1.04)	1		1.12 (0.99-1.26)	0.94 (0.76-1.15)
	Fear of economic loss due to infection	1	1.11 (1.00-1.23)	0.97 (0.79-1.19)	1		1.04 (0.95-1.13)	0.94 (0.80-1.10)
**Health behavior deterioration**								
	Decreased physical activity	1	*1.17 (1.10-1.25)*	*1.4 (1.24-1.58)*	1		*1.28 (1.22-1.35)*	*1.31 (1.21-1.43)*
	Changes in sleep duration	1	*1.62 (1.51-1.74)*	*2.28 (2.00-2.59)*	1		*1.71 (1.62-1.80)*	*2.15 (1.96-2.36)*
	Increased consumption of instant meals/soda	1	*1.47 (1.35-1.60)*	*1.64 (1.40-1.93)*	1		*1.36 (1.27-1.45)*	*1.6 (1.43-1.80)*
	Increased consumption of delivery food	1	*1.28 (1.19-1.39)*	*1.25 (1.07-1.45)*	1		*1.23 (1.15-1.31)*	*1.26 (1.12-1.41)*
	Increased alcohol drinking	1	*1.51 (1.33-1.71)*	*1.95 (1.58-2.42)*	1		*1.53 (1.36-1.73)*	*2.25 (1.88-2.70)*
	Increased smoking frequency	1	*1.64 (1.44-1.87)*	*2.55 (2.07-3.15)*	1		*1.53 (1.13-2.08)*	*2.71 (1.95-3.77)*

^a^Depression level was classified according to scores on the nine-item Patient Health Questionnaire.

^b^Logistic regression model adjusted for age, educational level, monthly household income, profession, region, heavy drinking, current smoking status, stress awareness, and subjective health status.

^c^Data were weighted to yield nationally representative estimates (total N=38,395,036).

^d^Reference category.

^e^Values in italics indicate a statistically significant association.

## Discussion

### Principal Findings

The purpose of this study was to determine the effect of an individual’s depression level on noncompliance precautionary behavior, COVID-19 fear, and health behavior deterioration, and to compare the different trends according to gender. Overall, there was a significant difference in noncompliance with precautionary behavior and health behavior deterioration according to the degree of mental health. Additionally, some components pertaining to COVID-19 fear were different according to depression status. Men and women with clinically relevant depression were more likely to not comply with precautionary behaviors. Furthermore, as mental health deteriorated, health behaviors declined, which tended to be worse in women.

In the case of men with clinically relevant depression, not performing regular ventilation and not wearing a mask indoors significantly increased. Similarly, women with clinically relevant depression were more than twice as likely to not ventilate regularly or not wear a mask indoors than were women who were not depressed. These results are similar to those of several previous studies [23,38,39]; however, in some important ways, they conflict with the results of other studies. For example, one study indicated that poor mental health increased preventive activities, while another suggested that there was no association between precautionary behaviors and mental distress in those with severe mental illness [40,41]. This dichotomy may also exist in relation to COVID-19 fear. Generally, feelings of distress such as anxiety and fear are known to increase precautionary behavior [23,42,43]. However, in our study, the participants tended to have decreased levels of fear as their mental health deteriorated. This association between fear and precautionary behaviors suggests that reduced fear may increase precautionary behavior noncompliance.

Generally, health behaviors decrease as mental health worsens. Notably, women with clinically relevant depression showed a dramatic increase in heavy alcohol consumption and smoking frequency. Previous studies have also reported that women’s drinking behaviors increased during the COVID-19 pandemic [44] and that depressive symptoms were also associated with an increase in heavy drinking [45].

However, an increase in social responsibility and a decrease in social support could worsen the mental health of women. In the context of the COVID-19 pandemic, women have been facing a variety of high-demand roles, including childcare and elder care, which most countries have traditionally imposed on them [17,46-48]. In addition, it is generally well known that social support has more of an effect on mental health for women than for men [49-51]. Restrictions on social gatherings, such as social distancing and guidelines that formed part of various quarantine measures, might have weakened the vital social support for women. In fact, various studies have provided empirical evidence that social distancing can affect mental health [52-54]. Moreover, research suggests that reduced physical activity due to social distancing may impair mental health [55]. Given that mental health is associated with smoking and drinking [56], changes in increased social responsibility and reduced social support can also worsen women’s mental health and lead to dramatic increases in drinking and smoking.

Overall, the trend of noncompliance with precautionary behaviors and a decrease in health behaviors according to worsening depression showed a greater change in women than in men. Although precautionary behavior compliance is the most important factor in responding to pandemic-related situations [57], health behaviors constitute the basic foundation for maintaining one’s physical and mental health, not only in the present but also in the future. Therefore, interventions that consider the degree of depression are necessary to alleviate depression, which could affect the implementation of precautionary behavior and health behavior. Additionally, further studies should examine different ways of implementing such interventions depending on gender.

### Study Limitations

This study had several limitations. First, the study did not consider time changes; it was difficult to ascertain causal relationships between the effects of depression and precautionary and health behaviors. In particular, since the KCHS data did not confirm the antecedent relationship between depression and COVID-19, it could not be confirmed whether the depression was due to COVID-19 or other factors. Second, in this study, the trends of precautionary behavior compliance, COVID-19 fear, and health behaviors according to the degree of depression were identified by gender, but it was not shown how vulnerable women were compared to men and their statistical significance. Third, because the data used in this research were collected in the early stages of the COVID-19 pandemic, the precautionary behavior compliance rate created a fairly high temporary bias. In other words, if the data were collected at the end of the pandemic, the results of precautionary or health behaviors may have changed. Finally, in light of the fact that a self-reporting survey was conducted, there is a possibility that measurement errors may have occurred because of the inherent difficulty in checking whether COVID-19 precautionary behaviors were actually being followed and whether health behavior had actually changed. However, this study was cross-sectional and used a representative sample from the entire Republic of Korea. Additionally, this study is valuable in that it examined how depressive symptoms and gender affect precautionary behaviors, even when social aspirations and interest in precautionary behaviors are high.

### Conclusion

In this study, we were able to identify how precautionary behavior compliance, COVID-19 fear, and health behaviors change according to the degree of depression and trends according to gender. We found that regardless of gender, people suffering from clinically relevant depression were highly likely to infringe on precautionary behaviors and deteriorate health behaviors. Additionally, this trend was more noticeable in women. These results can be useful for developing related interventions and for future studies that consider both mental status and gender.

## Appendix

Multimedia Appendix 1 Crude odds ratios (95% CIs) for failure to comply with precautionary behaviors, health behavior deterioration during the COVID-19 outbreak, and COVID-19–related fear according to depression levels.

## Abbreviations

AOR: adjusted odds ratio
IRB: institutional review board
KCHS: Korea Community Health Survey
PHQ-9: Patient Health Questionnaire-9

## Data Availability

The data set used in this study and more information about the data can be obtained from the official Korea Community Health Survey website [29].

## References

[1] (2022). WHO Coronavirus (COVID-19) Dashboard.

[2] Prem K, Liu Y, Russell TW, Kucharski AJ, Eggo RM, Davies N, Jit M, Klepac P, Centre for the Mathematical Modelling of Infectious Diseases COVID-19 Working Group (2020). The effect of control strategies to reduce social mixing on outcomes of the COVID-19 epidemic in Wuhan, China: a modelling study. Lancet Public Health.

[3] Thompson RN (2020). Epidemiological models are important tools for guiding COVID-19 interventions. BMC Med.

[4] Leung K, Wu JT, Liu D, Leung GM (2020). First-wave COVID-19 transmissibility and severity in China outside Hubei after control measures, and second-wave scenario planning: a modelling impact assessment. Lancet.

[5] Shanafelt T, Ripp J, Trockel M (2020). Understanding and addressing sources of anxiety among health care professionals during the COVID-19 pandemic. JAMA.

[6] Salari N, Hosseinian-Far A, Jalali R, Vaisi-Raygani A, Rasoulpoor S, Mohammadi M, Rasoulpoor S, Khaledi-Paveh B (2020). Prevalence of stress, anxiety, depression among the general population during the COVID-19 pandemic: a systematic review and meta-analysis. Global Health.

[7] Fancourt D, Steptoe A, Bu F (2021). Trajectories of anxiety and depressive symptoms during enforced isolation due to COVID-19 in England: a longitudinal observational study. Lancet Psychiatry.

[8] O'Connor RC, Wetherall K, Cleare S, McClelland H, Melson AJ, Niedzwiedz CL, O'Carroll RE, O'Connor DB, Platt S, Scowcroft E, Watson B, Zortea T, Ferguson E, Robb KA (2021). Br J Psychiatry.

[9] COVID-19 Mental Disorders Collaborators (2021). Global prevalence and burden of depressive and anxiety disorders in 204 countries and territories in 2020 due to the COVID-19 pandemic. Lancet.

[10] Voitsidis P, Nikopoulou VA, Holeva V, Parlapani E, Sereslis K, Tsipropoulou V, Karamouzi P, Giazkoulidou A, Tsopaneli N, Diakogiannis I (2021). The mediating role of fear of COVID-19 in the relationship between intolerance of uncertainty and depression. Psychol Psychother.

[11] Saravanan C, Mahmoud I, Elshami W, Taha MH (2020). Knowledge, anxiety, fear, and psychological distress about COVID-19 among university students in the United Arab Emirates. Front Psychiatry.

[12] Lin C, Broström A, Griffiths MD, Pakpour AH (2020). Investigating mediated effects of fear of COVID-19 and COVID-19 misunderstanding in the association between problematic social media use, psychological distress, and insomnia. Internet Interv.

[13] Taylor S, Asmundson GJG (2021). Negative attitudes about facemasks during the COVID-19 pandemic: the dual importance of perceived ineffectiveness and psychological reactance. PLoS One.

[14] Wang S, Quan L, Chavarro JE, Slopen N, Kubzansky LD, Koenen KC, Kang JH, Weisskopf MG, Branch-Elliman W, Roberts AL (2022). Associations of depression, anxiety, worry, perceived stress, and loneliness prior to infection with risk of post-COVID-19 conditions. JAMA Psychiatry.

[15] Kim HR, Kim J (2022). Stress, depression, and unhealthy behavior changes among patients with diabetes during COVID-19 in Korea. Healthcare.

[16] Brotto LA, Chankasingh K, Baaske A, Albert A, Booth A, Kaida A, Smith LW, Racey S, Gottschlich A, Murray MCM, Sadarangani M, Ogilvie GS, Galea L (2021). The influence of sex, gender, age, and ethnicity on psychosocial factors and substance use throughout phases of the COVID-19 pandemic. PLoS One.

[17] Hammarberg K, Tran T, Kirkman M, Fisher J (2020). Sex and age differences in clinically significant symptoms of depression and anxiety among people in Australia in the first month of COVID-19 restrictions: a national survey. BMJ Open.

[18] Zhang W, Walkover M, Wu YY (2021). The challenge of COVID-19 for adult men and women in the United States: disparities of psychological distress by gender and age. Public Health.

[19] Parker G, Brotchie H (2010). Gender differences in depression. Int Rev Psychiatry.

[20] Kiely KM, Brady B, Byles J (2019). Gender, mental health and ageing. Maturitas.

[21] Asmundson GJG, Taylor S (2020). How health anxiety influences responses to viral outbreaks like COVID-19: What all decision-makers, health authorities, and health care professionals need to know. J Anxiety Disord.

[22] Stickley A, Matsubayashi T, Sueki H, Ueda M (2020). COVID-19 preventive behaviours among people with anxiety and depressive symptoms: findings from Japan. Public Health.

[23] Olapegba PO, Chovwen CO, Ayandele O, Ramos-Vera C (2022). Fear of COVID-19 and preventive health behavior: mediating role of post-traumatic stress symptomology and psychological distress. Int J Ment Health Addict.

[24] Ahorsu DK, Imani V, Lin C, Timpka T, Broström A, Updegraff JA, Årestedt K, Griffiths MD, Pakpour AH (2022). Associations between fear of COVID-19, mental health, and preventive behaviours across pregnant women and husbands: an actor-partner interdependence modelling. Int J Ment Health Addict.

[25] Prowse R, Sherratt F, Abizaid A, Gabrys RL, Hellemans KGC, Patterson ZR, McQuaid RJ (2021). Coping with the COVID-19 pandemic: examining gender differences in stress and mental health among university students. Front Psychiatry.

[26] Simha A, Prasad R, Ahmed S, Rao NP (2020). Effect of gender and clinical-financial vulnerability on mental distress due to COVID-19. Arch Womens Ment Health.

[27] Xue B, McMunn A (2021). Gender differences in unpaid care work and psychological distress in the UK Covid-19 lockdown. PLoS One.

[28] Zhang W, Yang X, Zhao J, Yang F, Jia Y, Cui C, Yang X (2020). Depression and psychological-behavioral responses among the general public in China during the early stages of the COVID-19 pandemic: survey study. J Med Internet Res.

[29] (2020). Korea Community Health at a Glance 2020: Korea Community Health Survey (KCHS). Korea Disease Control and Prevention Agency.

[30] Thomas SL, Heck RH (2001). Analysis of large-scale secondary data in higher education research: potential perils associated with complex sampling designs. Res High Educ.

[31] About KLoSA. KEIS.

[32] Han MA, Kim HR (2021). Smoking behavior changes during COVID-19 among Korean adults. Am J Health Behav.

[33] Chung S, Lee E (2016). Patterns of time use across the life span in Korea: a latent class analysis and age and gender differences. Soc Indic Res.

[34] (2017). Korean Standard classification of occupations. Korean Standard Statistical Classification Portal.

[35] Han S, Ko Y, Moon JE, Cho YS (2021). Working hours are closely associated with depressive mood and suicidal ideation in Korean adults: a nationwide cross-sectional study. Sci Rep.

[36] Kroenke K, Spitzer RL, Williams JB (2001). The PHQ-9: validity of a brief depression severity measure. J Gen Intern Med.

[37] Lee GB, Jung SJ, Yiyi Y, Yang JW, Thang HM, Kim HC (2022). Socioeconomic inequality in compliance with precautions and health behavior changes during the COVID-19 outbreak: an analysis of the Korean Community Health Survey 2020. Epidemiol Health.

[38] Liang W, Duan Y, Shang B, Hu C, Baker JS, Lin Z, He J, Wang Y (2021). Precautionary behavior and depression in older adults during the COVID-19 pandemic: an online cross-sectional study in Hubei, China. Int J Environ Res Public Health.

[39] Imtiaz A, Khan NM, Hossain MA (2022). COVID-19 in Bangladesh: measuring differences in individual precautionary behaviors among young adults. Z Gesundh Wiss.

[40] Lee ATC, Cheng GWH, Lin C, Wong BHC, Lam LCW (2021). Do people with mental health problems have lower adherence to precautionary measures in COVID-19 pandemic? A cross-sectional observational study in Hong Kong. BMJ Open.

[41] Pinkham A, Ackerman RA, Depp CA, Harvey PD, Moore RC (2021). COVID-19-related psychological distress and engagement in preventative behaviors among individuals with severe mental illnesses. NPJ Schizophr.

[42] Harper CA, Satchell LP, Fido D, Latzman RD (2021). Functional fear predicts public health compliance in the COVID-19 pandemic. Int J Ment Health Addict.

[43] Kuper-Smith B, Doppelhofer LM, Oganian Y, Rosenblau G, Korn CW (2021). Risk perception and optimism during the early stages of the COVID-19 pandemic. R Soc Open Sci.

[44] Pollard MS, Tucker JS, Green HD (2020). Changes in adult alcohol use and consequences during the COVID-19 pandemic in the US. JAMA Netw Open.

[45] Nesoff ED, Gutkind S, Sirota S, McKowen AL, Veldhuis CB (2021). Mental health and economic stressors associated with high-risk drinking and increased alcohol consumption early in the COVID-19 pandemic in the United States. Prev Med.

[46] McGinty EE, Presskreischer R, Han H, Barry CL (2020). Psychological distress and loneliness reported by US adults in 2018 and April 2020. JAMA.

[47] Holingue C, Badillo-Goicoechea E, Riehm KE, Veldhuis CB, Thrul J, Johnson RM, Fallin MD, Kreuter F, Stuart EA, Kalb LG (2020). Mental distress during the COVID-19 pandemic among US adults without a pre-existing mental health condition: Findings from American trend panel survey. Prev Med.

[48] Stanton R, To QG, Khalesi S, Williams SL, Alley SJ, Thwaite TL, Fenning AS, Vandelanotte C (2020). Depression, anxiety and stress during COVID-19: associations with changes in physical activity, sleep, tobacco and alcohol use in Australian adults. Int J Environ Res Public Health.

[49] Wiklund M, Malmgren-Olsson E, Ohman A, Bergström E, Fjellman-Wiklund A (2012). Subjective health complaints in older adolescents are related to perceived stress, anxiety and gender - a cross-sectional school study in Northern Sweden. BMC Public Health.

[50] Van Droogenbroeck F, Spruyt B, Keppens G (2018). Gender differences in mental health problems among adolescents and the role of social support: results from the Belgian health interview surveys 2008 and 2013. BMC Psychiatry.

[51] Milner A, Krnjacki L, LaMontagne AD (2016). Age and gender differences in the influence of social support on mental health: a longitudinal fixed-effects analysis using 13 annual waves of the HILDA cohort. Public Health.

[52] Zarocostas J (2020). How to fight an infodemic. Lancet.

[53] Marroquín B, Vine V, Morgan R (2020). Mental health during the COVID-19 pandemic: effects of stay-at-home policies, social distancing behavior, and social resources. Psychiatry Res.

[54] Brooks SK, Webster RK, Smith LE, Woodland L, Wessely S, Greenberg N, Rubin GJ (2020). The psychological impact of quarantine and how to reduce it: rapid review of the evidence. Lancet.

[55] Jacob ST, Crozier I, Fischer WA, Hewlett A, Kraft CS, Vega MDL, Soka MJ, Wahl V, Griffiths A, Bollinger L, Kuhn JH (2020). Ebola virus disease. Nat Rev Dis Primers.

[56] Ferreira VR, Jardim TV, Sousa ALL, Rosa BMC, Jardim PCV (2019). Smoking, alcohol consumption and mental health: Data from the Brazilian study of Cardiovascular Risks in Adolescents (ERICA). Addict Behav Rep.

[57] (2022). Advice for the public: Coronavirus disease (COVID-19). World Health Organization.

