# Supplementary material for: Association of Depression With Precautionary Behavior Compliance, COVID-19 Fear, and Health Behaviors in South Korea: National Cross-sectional Study
Source: JMIR Public Health Surveill. 2023 Feb 22;9:e42677. doi: 10.2196/42677 (PMC9953990; doi:10.2196/42677)
Supplement: Multimedia Appendix 1 [file publichealth_v9i1e42677_app1.docx]

**Multimedia Appendix 1**

Crude odds ratios (95% CIs) for failure to comply with precautionary behaviors, health behavior deterioration during the COVID-19 outbreak, and COVID-19–related fear according to depression levels.^a,b^

| COVID-19–related questions | | Men (n=92,739) | | | Women (n=112,048) | | |
| --- | --- | --- | --- | --- | --- | --- | --- |
|  | | No depression^b^ (0-4) | Mild depression (5-9) | Clinically relevant depression (≥10) | No depression^b^ (0-4) | Mild depression (5-9) | Clinically relevant depression (≥10) |
|  | |  |  |  |  |  |  |
| **Failure to comply with precautionary behaviors** | | | | | | | |
|  | Not covering mouth while coughing | 1 | 1.46 (1.29-1.65) | 2.03 (1.66-2.47) | 1 | 1.58 (1.42-1.75) | 2.12 (1.83-2.45) |
|  | No proper ventilation | 1 | 1.75 (1.48-2.06) | 2.11 (1.58-2.81) | 1 | 1.8 (1.53-2.12) | 2.74 (2.21-3.40) |
|  | Not performing regular disinfection | 1 | 1.31 (1.24-1.39) | 1.59 (1.41-1.79) | 1 | 1.26 (1.20-1.31) | 1.39 (1.28-1.51) |
|  | Not wearing a mask indoors | 1 | 1.7 (1.19-2.40) | 2.15 (1.32-3.49) | 1 | 1.54 (1.10-2.14) | 2.2 (1.41-3.44) |
|  | Not wearing a mask when it was hard to maintain distance | 1 | 1.56 (1.23-1.97) | 1.66 (1.08-2.54) | 1 (ref) | 1.61 (1.27-2.05) | 2.04(1.46-2.85) |
|  | Not keeping the minimum recommended physical distance | 1 | 1.66 (1.46-1.88) | 1.64 (1.28-2.09) | 1 | 1.64 (1.47-1.81) | 1.8 (1.51-2.15) |
|  | Not refraining from visiting hospitalized patients | 1 | 2.07 (1.63-2.64) | 2.32 (1.44-3.72) | 1 | 1.46 (1.17-1.82) | 1.73 (1.23-2.44) |
|  | Not refraining from going out | 1 | 1.79 (1.52-2.10) | 1.86 (1.42-2.42) | 1 | 1.65 (1.43-1.89) | 1.48 (1.17-1.86) |
| **COVID-19–related fears** | | | | | | | |
|  | Fear of infection | 1 | 1.07 (0.97-1.17) | 0.83 (0.70-0.98) | 1 | 1.17 (1.06-1.28) | 0.85 (0.73-0.98) |
|  | Fear of dying from infection | 1 | 1.13 (1.06-1.20) | 1.27 (1.13-1.44) | 1 | 1.2 (1.14-1.26) | 1.23 (1.12-1.35) |
|  | Fear of public criticism | 1 | 0.96 (0.88-1.04) | 0.84 (0.72-0.98) | 1 | 1.11 (1.03-1.20) | 0.99 (0.79-1.02) |
|  | Fear of a family member getting infected | 1 | 1.25 (1.09-1.43) | 1.05 (0.85-1.31) | 1 | 1.32 (1.18-1.48) | 1.2 (0.99-1.47) |
|  | Fear of economic loss  due to infection | 1 | 1.32 (1.20-1.47) | 1.35 (1.10-1.64) | 1 | 1.24 (1.14-1.36) | 1.32 (1.14-1.54) |
| **Health behavior deterioration** | | | | | | | |
|  | Decreased physical activity | 1 | 1.26 (1.19-1.33) | 1.49 (1.33-1.67) | 1 | 1.34 (1.28-1.40) | 1.39 (1.28-1.50) |
|  | Changes in sleep duration | 1 | 1.9 (1.77-2.03) | 3.02 (2.67-3.42) | 1 | 1.93 (1.84-2.03) | 2.64 (2.42-2.87) |
|  | Increased consumption of instant meals/soda | 1 | 1.68 (1.56-1.81) | 1.84 (1.59-2.14) | 1 | 1.45 (1.37-1.54) | 1.7 (1.54-1.88) |
|  | Increased consumption of delivery food | 1 | 1.37 (1.28-1.46) | 1.21 (1.06-1.38) | 1 | 1.24 (1.18-1.30) | 1.23 (1.12-1.34) |
|  | Increased alcohol drinking | 1 | 1.96 (1.74-2.20) | 2.62 (2.15-3.18) | 1 | 1.92 (1.72-2.14) | 3.09 (2.64-3.61) |
|  | Increased smoking frequency | 1 | 2.55 (2.27-2.86) | 4.87 (4.06-5.84) | 1 | 3.85 (2.97-5.00) | 14.37 (10.92-18.91) |

^a^Depression level was classified according to scores on the nine-item Patient Health Questionnaire (PHQ-9).

^b^Data were weighted to yield nationally representative estimates (total N=38,395,036).

^c^Reference category.
